# *Capnocytophaga canimorsus* as Cause of Fatal Sepsis

**DOI:** 10.1155/2019/3537507

**Published:** 2019-05-23

**Authors:** Moritz Hundertmark, Tatjana Williams, Anja Vogel, Maria Moritz, Peter Bramlage, Nikolaos Pagonas, Oliver Ritter, Benjamin Sasko

**Affiliations:** ^1^Department of Internal Medicine I, University Hospital of Würzburg, Würzburg, Germany; ^2^Oxford Centre for Clinical Magnetic Resonance Research, John Radcliffe Hospital, University of Oxford, Oxford, UK; ^3^Department of Internal Medicine I–Cardiology, Brandenburg Medical Faculty, Brandenburg, Germany; ^4^Institute for Pharmacology and Preventive Medicine, Cloppenburg, Germany

## Abstract

A rare consequence of dog bites is the infection with *Capnocytophaga canimorsus*, and only a few cases have been documented. We describe a 41-year-old, formerly healthy woman who died from septic shock and multiorgan failure. It is the first case of a young individual without obvious immunosuppression.

## 1. Introduction

Although dog is considered to be “man's best friend,” dog bites are a common reason for admission to the emergency department (ED) [1]. The majority of cases do not imply severe trauma or illness, and localized infections are often due to *Pasteurella* spp. subspecies or a mix of different germs. The human infection with Gram-negative *Capnocytophaga canimorsus* (Latin = dog bite) gain more and more attention. Sepsis due to *C. canimorsus* is extremely rare, and only few cases have been described worldwide [2]. Interestingly, 40% of infections had no identifiable risk factor [3]. Recent research presents a possible mechanism for the endotoxicity of this Gram-negative bacterium and its ability to avoid the innate immune response [4].

A study in Japan found 74% prevalence in the oral cavity of dogs [5]. Though it is also found in the oral cavity of cats, there are only few reported infections from cat bites [6], possibly due to lesser tissue damage caused by cat bites. This fastidious, Gram-negative, slow-growing bacterium is associated with severe infections, especially in immunocompromised patients (postsplenectomy and chronic alcohol-abuse being most common). The mortality among *C. canimorsus* infections varies between 25 and 30% and is doubled in patients with septic shock [7, 8].

## 2. Case Presentation

A 41-year-old woman was admitted to our ED with a facial dog bite that occurred 4 days before. Her dog was sitting in her lap when, without an obvious reason, he bit her in the face. Because of the initial mild complaints without visible bleeding, the patient did not seek medical attention at the time.

**Figure 1:**
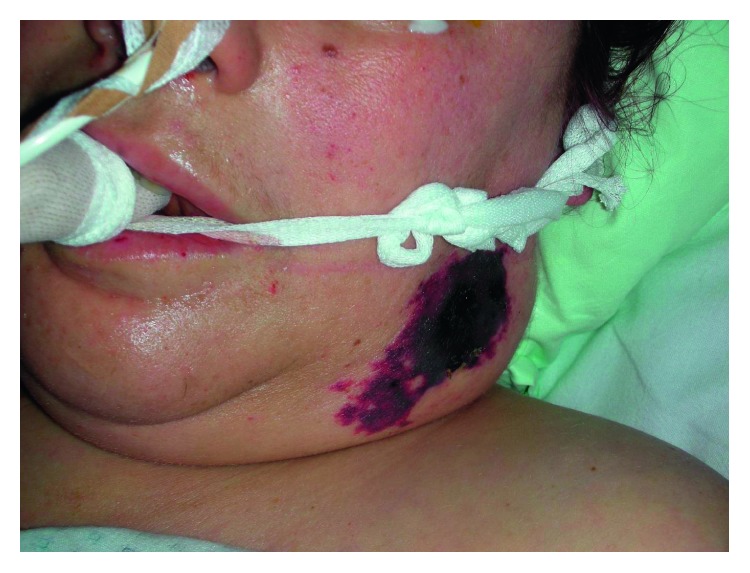
Necrotic facial tissue after dog bite to the left submandibular region.

**Figure 2:**
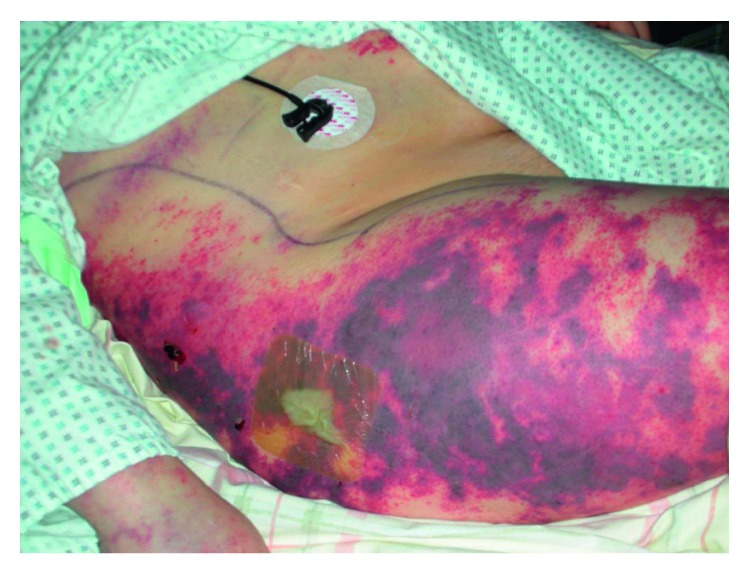
Petechial bleeding due to DIC on the right flank and thigh.

**Figure 3:**
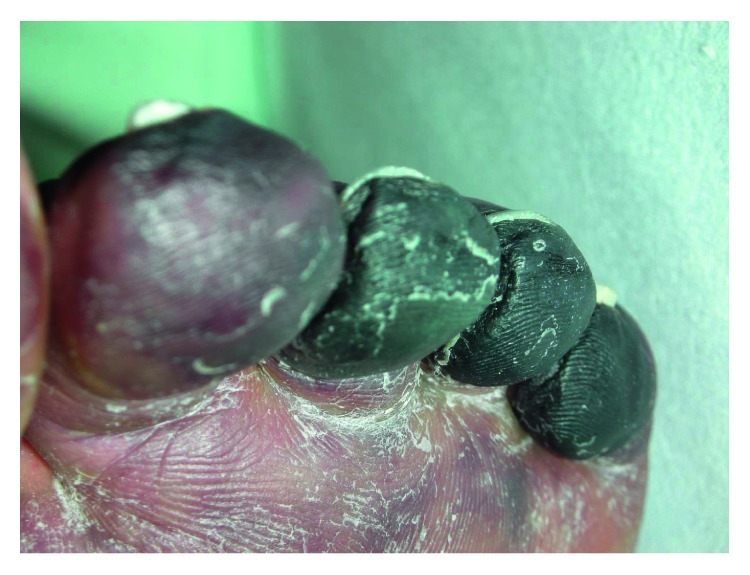
Necrotic left toes as a sign of septic shock due to infection with *C. canimorsus*.

Only three days later, she began to feel affected and developed fever as well as a rash with marbled skin on her whole back, her extremities, and her face (Figure 1). The medical history included a chronic alcoholism with long-term abstinence and obesity. She was admitted to the next general hospital where she showed signs of systemic inflammatory response syndrome (SIRS) with tachypnea (30/min), fever (39°C), tachycardia (140/min), thrombopenia, and leucocytopenia as well as hypoglycemia (50 mg/dl). There was no evidence of chest or abdominal infection. Because of progressive hemodynamic instability under treatment with norepinephrine, she was transferred to our university hospital. Endotracheal intubation and mechanical ventilation were initiated shortly after admission, and the initial antibiotic treatment with ciprofloxacin and amoxicillin/clavulanic acid was escalated to fosfomycin, clindamycin, and meropenem. After initial fluid resuscitation, the hemodynamic therapy was continued with norepinephrine and goal-directed infusion therapy. Multiorgan failure included the circulatory system, renal and hepatic insufficiency, and disseminated intravascular coagulation (Figure 2) with clear signs of purpura fulminans and necrosis to both feet (Figure 3). Despite high doses of antibiotics and optimal sepsis treatment, there was no sign of stabilization within the following days. Due to progressive acute renal failure in septic shock, hemodialysis (CVVHDF) was necessary for 10 days and had to be continued intermittently. Twelve days after the beginning of treatment, there was 16S-RNA verification via PCR for *C. canimorsus*. Despite all efforts to cultivate this germ before beginning antibiotic treatment in multiple blood cultures, the detection could not be achieved. In the following weeks, the patient developed secondary infection with PAS-positive yeast and *Enterococcus faecium*. A CT showed cerebral and hepatic septic lesions, whereas no endocarditis was seen in repeated transesophageal echocardiograms. Additionally, a surgical tracheostomy was performed. Because of relevant bleeding signs and a positive DIC score, recurrent transfusions (RBC, 3 WBC, and FFP) were necessary. Constant surgical treatment including several necrosectomies of facial wounds was vital.

Finally, the patient sustained massive hemodynamic instability and suffered cardiac arrest. No resuscitative efforts were undertaken due to the alleged patient's will in accordance with the patient's relatives. The patient died because of massive septic shock.

## 3. Discussion

The vast majority of animal bites do not lead to ED admission [1]. While most infections due to animal bites are caused by dogs, only few patients develop sepsis. *C. canimorsus* has an exceptional status because it is generally associated with dramatic infections and a high mortality rate [5]. First described in 1976 as an unidentified Gram-negative bacterium in a patient with meningitis, the name was given 13 years later, mostly to the usual mode of transmission [9]. Early identification of possible harmful infections is difficult because *C. canimorsus* special lipopolysaccharide (LPS) composition enables it to escape the innate immune system at first [10]. This is a possible explanation why even serious infections seem to begin with mild symptoms in most cases.

The incubation period before the onset of symptoms ranges from 1 to 7 days, while patients typically present with fever, shortness of breath, malaise, and often localized cellulitis at site of infection [7]. Generalized infection can lead to DIC as well as endocarditis, meningitis, osteomyelitis, peritonitis, and, like in our case, purpura. Due to hemorrhagic infarction of small subdermal vessels, patients develop the typical discoloration of the skin.

The differential diagnosis of the cutaneous lesions in this case includes frostbite on the basis of the necrotic toes, different types of systemic vasculitis, as these may be the potential cause for the diagnosed DIC leg lesions, as well as infections with other pathogens. The clinical presentation of the impressive and progressive dermatologic manifestations is suggestive of a process that is both local and systemic. Due to the combination of the dermatological with severe systemic symptoms, as well as the history of dog bite, and the successful PCR-based proof for *C. canimorsus*, the diagnosis of a causative infection with this germ was made.

Methods for detection of *C. canimorsus* include bacterial culture of blood, blood smears, and new diagnostic tools like 16S-RNA detection by PCR [8]. Since the germ is slow growing and some strains do not grow at all, it is important to take a possible infection with *C. canimorsus* into consideration when a patient presents with a dog bite. Because of the fast and progressive course of the infection, an antibiotic therapy has to be started before the bacteriological finding confirms the suspicion [11]. The cultivation of the germ in blood medium is difficult and tedious [12]. In contrast to the difficult detection of the pathogen, it has a good susceptibility profile. *Capnocytophaga* species are susceptible against *ß*-lactams [13]. Therefore, first-line treatment of proposed *C. canimorsus* infections are *ß*-lactams, while multiple other antibiotic agents have been proven effective [12]. The most important step in the initial management of dog bites includes painstaking wound preparation. While simple lacerations may be closed primarily, the majority of dog bites with severe trauma may heal by secondary intention since foreign material may increase the risk of infection [14]. Antibiotic prophylaxis is difficult because most infected dog bite wounds host polymicrobial organisms [15]. *ß*-Lactamase production is becoming increasingly common among *Capnocytophaga* spp., as in other bacteria [16]. The use of amoxicillin in combination with the *ß*-lactamase inhibitor clavulanate potassium represents the first-line oral therapy. Intravenous treatment includes doxycycline, clindamycin, meropenem, and cefuroxime [17]. The best prophylaxis for septic infections is proper treatment at the earliest time possible. Apparent signs of wound infection should lead to preservation of blood cultures and surgical consultation for possible operative exploration. In case of infective signs, antibiotics should be administered intravenously for a course of 14 days. Tetanus immunization status and postexposure prophylaxis for rabies should also be addressed.

Taken together, this case underlines the significance of correct risk evaluation in patients with dog bites. *C. canimorsus* is a fastidious Gram-negative germ, which might account for a large number of infections. It can cause severe illness not only in immunocompromised patients but also in other patients. We showed that chronic alcohol consumption in the past may be a single risk factor for the development of septic bacteraemia with *C. canimorsus* after dog bites.
